# Naturally occurring *Neisseria gonorrhoeae* can have large deletions in housekeeping gene *abcZ*, making them untypable with multilocus sequence typing

**DOI:** 10.1099/mgen.0.000889

**Published:** 2022-09-22

**Authors:** Hilde Synnøve Vollan, Dominique A. Caugant, Vegard Eldholm, Kristian Alfsnes, Nadia Debech, Ola Brynildsrud

**Affiliations:** ^1^​ Division of Infection Control, Norwegian Institute of Public Health, Oslo, Norway; ^2^​ Department of Community Medicine, Faculty of Medicine, University of Oslo, Oslo, Norway; ^3^​ Food Safety and Infection Biology, Norwegian University of Life Science, Ås, Norway

**Keywords:** abcZ, MLST, *Neisseria*, operon, WGS

## Abstract

The *abcZ* gene is an essential housekeeping gene in all the *Neisseria* species. It is one of the seven genes used for multilocus sequence typing (MLST) this genus. It encodes the cytosolic component of an ATP-binding cassette (ABC) transporter complex of unknown function. We report here the finding of a strain of *Neisseria gonorrhoeae* with a 485 base pair deletion in the 5′ region of the *abcZ* gene that truncates the protein product from 636 amino acids to 89 amino acids. A second open reading frame (ORF), encoding the latter 388 amino acids of the *abcZ* gene, was predicted downstream. The deletion will affect MLST profiling; interrogation of genomic sequences from PubMLST revealed that this isolate is not an anomaly. Deletions in *abcZ* were identified in 256 *Neisseria* genomes, roughly 0.6% of isolates. Furthermore, these deletions could leave the *abcZ* gene in a pseudogenized state. Our strain, isolated from a patient with symptoms of gonorrheal infection, nevertheless behaved normal in terms of growth and *in vitro* phenotypic properties.

## Data Summary

The Sequence Read Archive at National Centre for Biotechnology Information Search database (NCBI SRA) Accession ID SRR3360624 was used. This strain has PubMLST id 48 638. Furthermore, we discuss PubMLST gene accessions NEIS1015/NGO0870 (AbcZ) and NEIS1016/NGO0869 (DedA). Finally, we use the *Escherichia coli* AbcZ with Accession ID AF-P0ABP6-F1 in the AlphaFold Protein Structure Database. The NCBI accession number for *Neisseria gonorrhoeae* strain 659 717 is SAMEA6534160. Oxford Nanopore GridION reads are separately available using accession ERR8050709.

*Neisseria gonorrhoeae* core genome MLST v 1.0 scheme in PubMLST was used to find the closest genomic homolog [1]. FGENESB [2] was used to study the AbcZ operon, while YASARA Structure and WHATIF twinset software v19.7.5 [3] was used to create an AbcZ model using the hm_build and hm_refine macros [4, 5], while the PDB server [6] (https://www.rcsb.org/) was used to search for the closest AbcZ and DedA homologs. I-Tasser [7] was used to model the DedA membrane protein. SWISS-MODEL Structure Assessment [8] was used to assess the models. The pSortB v.3.0 [9] was used to predict DedA location. The DedA and AbcZ models can be found at https://figshare.com/projects/AbcZ/133386.

**Impact Statement**

Large deletions in the *abcZ* housekeeping gene means that multilocus sequence typing is not always possible in *N. gonorrhoeae *. A truncation of the gene that resulted in the deletion of one of the two nucleotide-binding domains (NBDs) had no obvious *in vivo* or *in vitro* handicap. A thorough search of more than 40,000 publicly available genomes revealed that deletions in the 5' or 3' regions of *abcZ* were present in roughly 0.6% of all *Neisseria* spp, however all mutants had at least one NBD intact. iThese findings imply that possession of both NBDs are not essential under all growth conditions.

## Introduction

*Neisseria gonorrhoeae* is an obligate pathogen commonly colonizing human genitalia as well as mucosa in the oropharynx and rectum [10]. Colonization and proliferation on these membranes causes the sexually transmittable disease (STD) gonorrhoea, the world’s second most common STD with an estimated 87 million new cases world-wide in 2016 [11].

*N. gonorrhoeae* is commonly classified using multilocus sequence typing (MLST), which assigns sequence types based on nucleotide differences in seven different housekeeping genes. The seven genes in this MLST scheme are chosen because they display moderate mutation rates and because they are known to be 'housekeeping' genes, i.e. essential for proper function of the pathogen. One requirement is that a strain has a non-empty allele at all seven loci. Originally developed in 1998, the MLST scheme for *Neisseria* spp. is the oldest known MLST scheme [12, 13]. It was originally based on six housekeeping genes, but a seventh locus, *fumC*, was added in 2000 [14].

One locus that has always been part of the scheme is the *abcZ* gene that putatively encodes an ATP-binding cassette (ABC) transporter subunit. ABC transporters are ubiquitous membrane proteins that use ATP to translocate a wide variety of substrates across cell membranes. ABC transporters can both function as importers that bring nutritional substrates into the bacterial cell, and as exporters that bring drugs, toxins, catabolites, and other unwanted molecules out of the cell. Bacteria have many different ABC transporters with individual substrate affinity, but they all share a similar architecture consisting of two cytoplasmic nucleotide-binding homodimer domains (NBDs) two homodimer transmembrane domains (TMDs). The NBDs and the TMDs can either be encoded by different genes or by one, fused, gene. In both cases the NBDs form a sandwich dimer in an antiparallel fashion, and the binding of one or two ATP molecules is facilitated at the interface between both subunits. Similarly, two TMD subunits are complexed with the ATPase in the formation of the translocation pathway. ABC type ATPases contain conserved motifs involved in ATP binding, dimerization and hydrolysis. Among these are Walker A (GxxGxGKST, where x can be any amino acid), Q-loop (xxQxx/YxxQ), and, as well as signature sequence (LSGGQ/E), Walker B motifs (φφφφDE, where φ indicate a hydrophobic amino acid), d-loop (xxLD) and a Switch sequence motif (xxHxx) [15–18].We describe here the finding of a strain with a large deletion in the *abcZ* gene, removing one of two NBDs. The strain, named 659 717 or ∆*abcZ*, was isolated from a patient with clinical symptoms of gonorrhoea. Further scrutiny of publicly available genomes reveals similar but distinct NBD-engulfing deletions in several hundred strains.

## Methods

### Isolate collection and wet lab work

The mutant strain 659 717 was first isolated at a primary healthcare institution from a patient with clinical symptoms of gonorrhoea in 2019. It was subsequently submitted to the Norwegian Institute of Public Health (NIPH) reference laboratory for gonococci. The isolate was grown overnight at 37 °C in an atmosphere of 5 % CO_2_ on chocolate blood agar for DNA extraction. Antibiotic susceptibility testing (AST) was done on GC agar base supplemented with 1 % IsoVitaleX and 1 % haemoglobin. To examine potential resistant isolates the minimum inhibitory concentrations (MIC) of antibiotics were determined using E-tests according to the manufacturer’s instructions (bioMérieux, Marcy-l'Étoile, France). The antibiotics tested were ciprofloxacin (CIP), ceftriaxone (CRO), cefixime (CFM), azithromycin (AZM), penicillin G (PCN), spectinomycin (SPX) and tetracycline (TET). The minimum inhibitory concentration (MIC) values were as follows: CIP 2, CRO 0.002, CFM 0.016, AZM 0.25, PCN >32, SPX 8, TET 16.

### Whole-genome sequencing

For the short-read sequencing DNA was extracted using MagNA Pure 96 (Roche Life Science). DNA sequencing libraries were created using KAPA HyperPlus kits (Roche Life Science) with NEXTflex DNA barcodes (Bioo Scientific) following the manufacturer’s instructions. DNA libraries were sequenced on the Illumina MiSeq platform three times and the Illumina NextSeq platform once. V3 600-cycles reagent kits (Illumina) were used according to the manufacturer’s instructions.

For long-read sequencing, high molecular weight DNA was extracted from cultured bacteria using the genomic-tip 20 G^−1^ kit and buffer set (Qiagen). Sequencing libraries were prepared with the rapid barcoding kit (SQK-RBK001) and 1D reads were generated on the GridION platform using the R10 (FLO-MIN106) flow cell.

### Bioinformatic analyses

For assembly we employed a hybrid approach implemented in Unicycler v0.4.7 [19], using both long Oxford Nanopore reads and short, accurate Illumina reads. This resulted in one circular genome of 2 219 441 bp (The gonococcal chromosome) and three plasmids: A 43 016 bp plasmid (*tetM*-associated plasmid, Dutch type), a 5 600 bp plasmid (beta-lactamase-carrying plasmid Africa/pJD-5), and a 4 207 bp plasmid (Cryptic plasmid pJD1, ostensibly present in 96 % of all gonococci [20]). We used the *Neisseria gonorrhoeae* core genome MLST v 1.0 scheme as implemented in PubMLST to identify the closest whole-genome sequenced relative to the mutant strain 659 717. The closest publicly available sequence was SRR3360624 [PubMLST id 48 638], a sequence type of ST-1588 strain isolated in Brighton, UK in 2005 [21], with an allelic distance of 313. (The cgMLST scheme contains a total of 1605 loci). SRR3360624 and 659 717 are identical at all MLST loci except *abcZ* and both have the PorA 18–10,43; FetA 4–7 finetype. The ~1640 nucleotides upstream and ~7880 nucleotides downstream sequence of the *abcZ* gene sequences was extracted and aligned for both genomes.

The frequency of *abcZ* deletions in the PubMLST database was examined in detail to find the frequency of larger deletions in *abcZ*. We screened the entire database for A) the presence of one of the three truncated NEIS1015 (full-length *abcZ*) alleles: 153, 165 or 594, and B) the lack of any NEIS1015 allele in isolates with a total sequence bin length of at least 500 000 nucleotides. (Essentially, any isolate with WGS data). We excluded isolates that had missing alleles for any of the following reasons: 1) The truncated *abcZ* gene was located at the end of a contig; 2) the predicted reading frame was associated with a full-length protein that had not yet received an allele number; 3) isolates with non-frameshift indels that resulted in slightly shorter or longer proteins; 4) isolates with no significant partial matches to *abcZ* (defined as at least 80 % nucleotide identity to any allele and with a length of at least 300 nucleotides). Isolates excluded for reasons 1 and 4 could potentially have truncations in *abcZ*, but are more likely products of poor sequence quality.

The NGFG_00858 gene in *N *. *gonorrhoeae* strain MS11 gene is according to Remmele *et al.* an essential gene [22]. This gene encodes for the AbcZ protein (with GenBank accession ID EEZ47691.1) and has a 99.69 % sequence identity to the intact protein sequence from *N *. *gonorrhoeae* strain 48 638 (File S2). However, it is unclear from the analysis of Remmele *et al.* whether partial *abcZ* truncations, i.e*.* mutants with one ATPase region intact, could have minimally altered function.

### Homology protein structure modeling

The AbcZ protein model was constructed using the homology modelling macro in the YASARA Structure and WHATIF twinset software v19.7.5 (using the hm_build.mcr macro) [3, 23]. The model was then optimized with a short MD simulation running the YASRARA refine macro [4, 5]. The YASARA Structure and WHATIF twinset software was also used for all structure analyses and visualisations. Structural alignments were performed using YASARA’s built in MUSTANG module [24]. The AbcZ-1 and AbcZ-2 models were constructed using I-Tasser [25].

A gene that belongs to the TMD DedA superfamily is located next to the *abcZ* gene in *N. gonorrhoeae *. It is predicted that the *dedA* and *abcZ* genes are in the same operon and could thus be interacting domains in the membrane. Due to low homology to existing structures, neither the YASARA software nor blast homology search at the PDB server [6] (latest search date: 11 January 2022) could find a structural homologue to the DedA sequence. The closest solved structure is the CusA efflux pump (PDB ID 3K07), but the sequence identity to DedA is less than 25 %. The online servers pSortB v.3.0 (9) and I-Tasser [25] were used to create topology, 3D structure models and estimate location of the proteins in the bacterial cell (e.g*.* if DedA is located in the membrane or is more likely to be a cytosolic protein) [7].

**Fig. 1.**
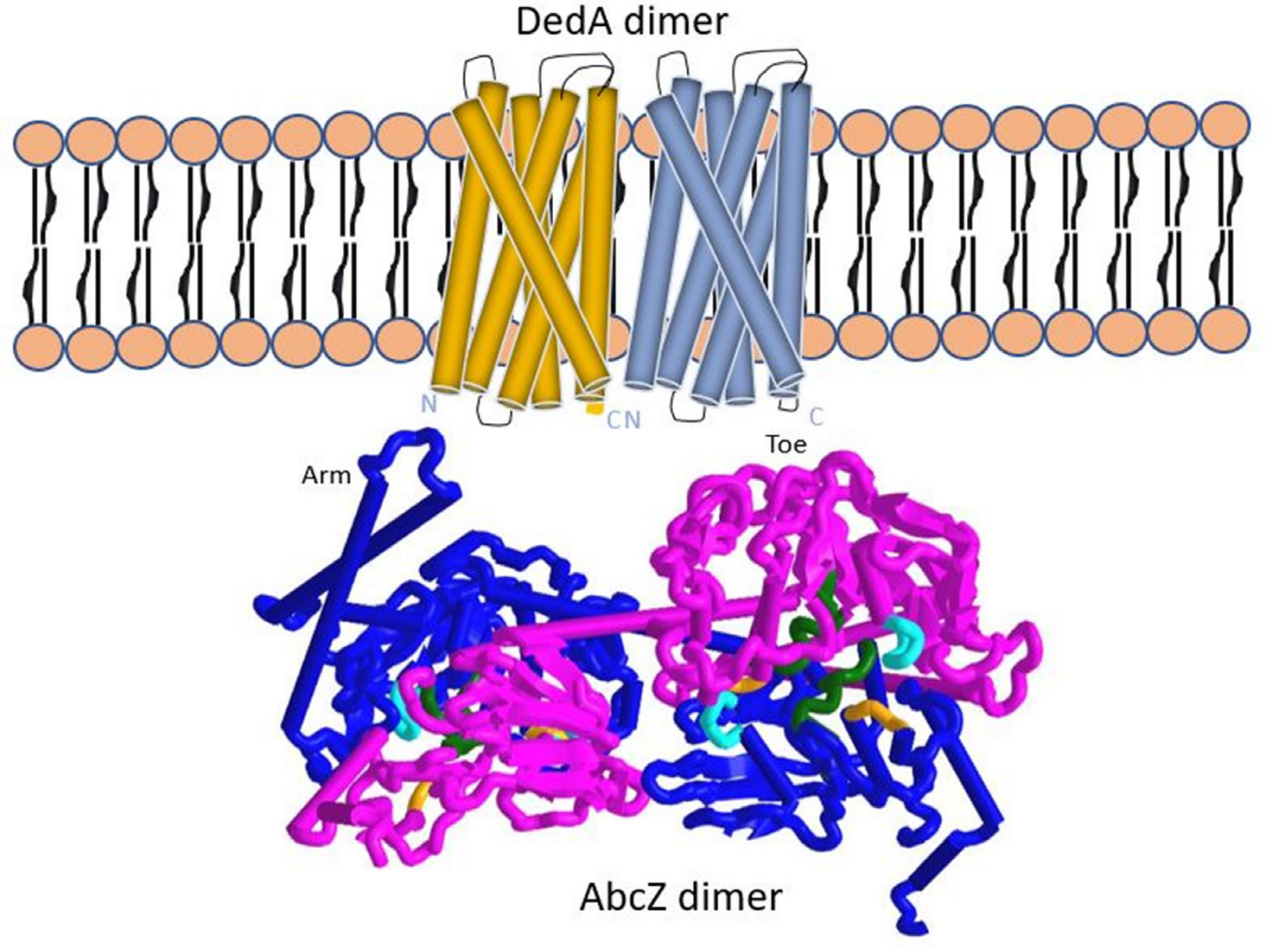
Theoretical TM DedA homodimer interacting with AbcZ homodimer. This tube style TM-helices were visualized coloured by secondary structure using r3dmol. DedA models were created using the I-Tasser server, while the AbcZ models were created using YASARA-WHATIF twinset.

The two homology models were cleaned using YASARA Structure and WHATIF twinset software v19.7.5 and assessed by the SWISS-MODEL structural assess server [8]. The AbcZ cartoon visualization in Fig. 1 was created using iCn3d. Preliminary interaction between DedA and AbcZ in this figure based on Boël *et al.* [26].

## Results

**Fig. 2.**
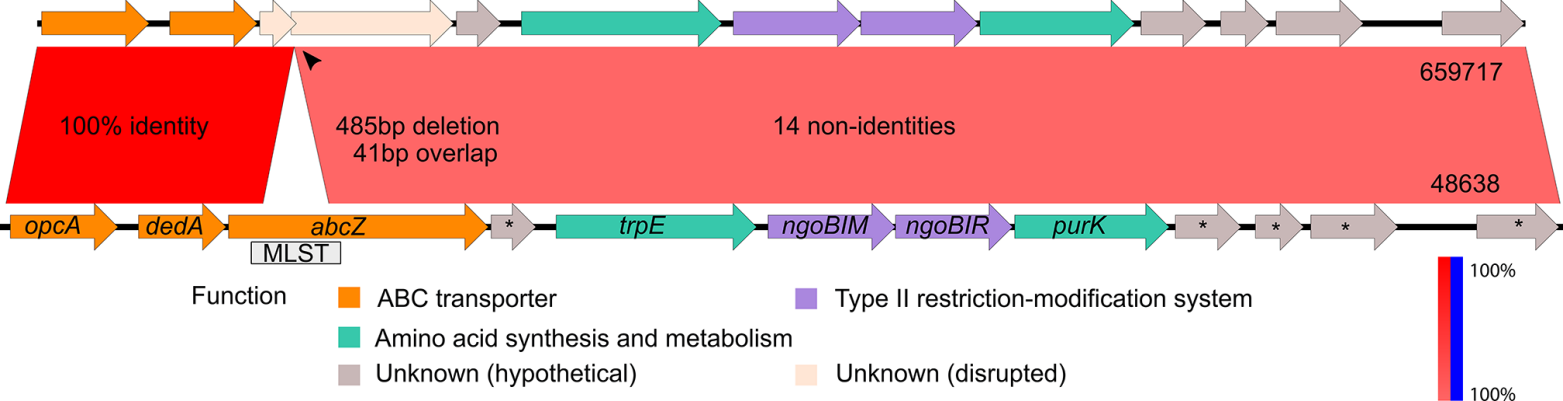
Genomic neighbourhood of *abcZ* in the ∆*abcZ* strain 659 717 and the closest related publicly available genome, SRR3360624 [PubMLST id 48 638]. In 659 717, the *abcZ* ORF is disrupted by a 485 bp deletion, resulting in two smaller ORFS, *abcZ-1* and *abcZ-2*. Immediately upstream of *abcZ* is the *dedA* gene. These two genes are in the same operon and share a promotor and a terminator. Genetically, the region is highly conserved, with 100 % sequence identity upstream of the deletion (*opcA::abcZ-1*) and just three non-identities downstream in the *abcZ-2::purK* region.

The ∆*abcZ* mutant strain 659 717 had a 485 bp deletion in the *abcZ* gene (NEIS1015; NGO0870) when compared to its closest relative, introducing a premature stop (opal) codon 270 nucleotides downstream from the 5′ terminal. This truncates the length of the AbcZ protein from 636 amino acids to 89. We will call this ORF *abcZ-1*. An alternative start codon TTG, located 23 nucleotides upstream of the deletion opens a secondary ORF containing the latter 398 amino acids of the AbcZ protein. We will call this ORF *abcZ-2*. Fig. 2 shows the genomic neighbourhood in the ∆*abcZ* strain and the closest related publicly available sequence.

**Fig. 3.**
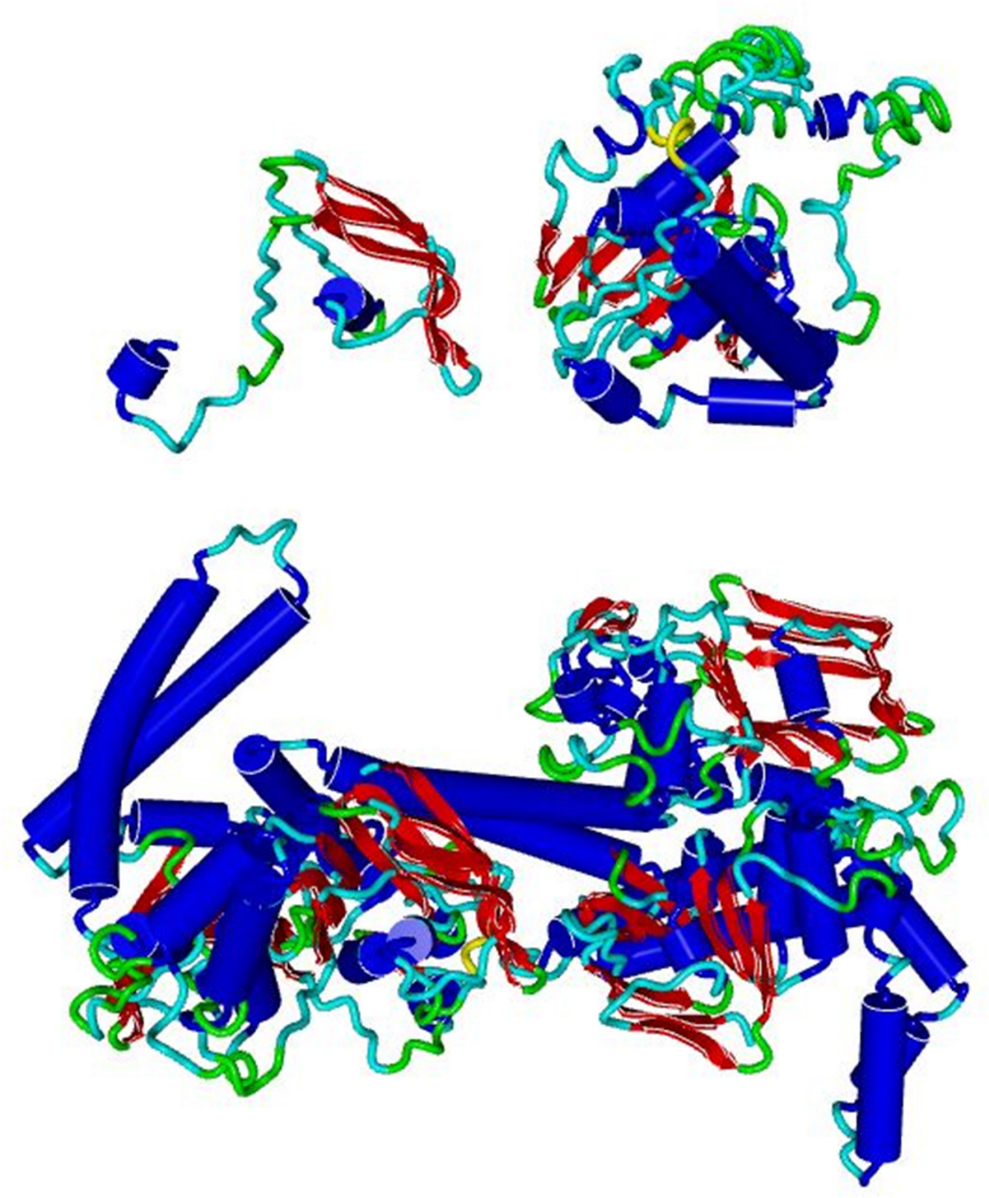
The top figures illustrate AbcZ-1 and AbcZ-2, while the bottom figure illustrate the AbcZ dimer model. Both are visualized using YASARA-WHATIF.

The intact AbcZ model and the two predicted ORFs AbcZ-1 and AbcZ-2 are mapped in Fig. 3b. These are hybrid models, but the majority of the intact AbcZ model was based on the solved *E. coli* EttA structure (PDBid 4FIN). EttA is an ATPase that binds to the ribosome, and deficient EttA proteins inhibit protein synthesis [26]. Although the closest solved structure to AbcZ (strain 48638) is *E. coli* EttA (with 38 % sequence identity to PDBid 7MU0), it is more likely that AbcZ is an ATPase coupled to a membrane and not involved in protein synthesis.

To understand how frequent deletions in the *abcZ* gene are, we examined the entire collection of *Neisseria* genomes from PubMLST. A total of 42 819 genomes were eligible after requiring a total sequence bin length of at least 500 000 bp (see Methods). A total of 256 isolates were found to have truncated *abcZ* reading frames, the intact reading frames ranging in length from 528 to 1848 nucleotides. Seventy genomes had truncations of the 5′ end of the reading frame, and 186 of the 3′. All mutants had at least one ATPase region intact. The *abcZ* mutant genes displayed a high degree of heterogeneity, indicating that truncation of the gene is not a unique event. Furthermore, this has occurred in different species, with 81 being *N. meningitidis *, 75 *N. gonorrhoeae *, one *N. lactamica* and one *N. cinere* a. If these isolates are representative of the *Neisseria* genus, it would indicate that roughly 0.6 % of all *Neisseria* have major truncations in the *abcZ* gene. (Visual overview of all unique deletions, *N*=108, can be found in Fig. S1).

The conserved motifs defined in the introduction for AbcZ are highlighted in Fig. S2. The deleted region is in the α-helical domain of the NBD monomer, a region that contains the ABC transporter signature motif LSGGQ (found in the α-helical domain of the NBD intact monomer in AbcZ). This motif is involved in ATP catabolism together with the so-called P-loop of the catalytic core domain in the opposing NBD monomer. Without the LSGGQ signature motif, which the AbcZ-2 lacks, the monomers might not interact which could result in a broken transporter. The Q-loop of the α-helical domain is also in the deleted region of the *abcZ* gene in the mutant strain. This is the part that interacts with the TMDs in the assembled ABC transporter. Furthermore, the AbcZ-1 and AbcZ-2 from the resulting ORFs are missing a fragment of 149 amino acids compared to the intact AbcZ. Our predictions show that this could result in two protein fragments that may be degraded by the bacterial protein control system. No homologous genes were found elsewhere in the genome sequence. The AbcZ model is shown in Fig. 3a. In Fig. 3b, AbcZ-1, AbcZ-2 and the deleted region is visualized.

The TMD subunit is most likely encoded by the *dedA* gene (NEIS1016; NGO0869), which is located 19 bp immediately upstream of the *abcZ* gene. The *dedA* and the *abcZ* ORFs are likely transcribed in a single operon since they are located closely together on the same strand, share a promotor closely upstream of *dedA*, a terminator downstream of *abcZ*, and because exactly two copies of each monomer are required to assemble an ABC transporter. The *dedA* gene in the ∆*abcZ* strain shares 100 % sequence identity to that of known functional alleles and thus appears to be intact. This indicates that the *abcZ* deletion is a recent event, as the TMDs of ABC transporters are not very conserved genetically even when they can actually junction with a functional NBD protein. Although it is annotated as a SNARE-associated Golgi protein by most commonly used protein databases, it is known that *dedA*-family proteins can function as membrane transporter complexes [27].

## Discussion

We have found that it will sometimes be impossible to get a complete MLST profile for *Neisseria* spp. since deletions several hundred amino acids in length in the *abcZ* gene, including over the MLST typing region and across NBDs, seem to be compatible with normal bacterial behaviour and growth. This appears to happen rarely (seen in <1 % of isolates), but plenty of deletion events have occurred independently as evident from our screening of the global collection in PubMLST.

The ∆abcZ strain was not noteworthy in terms of manifestation of disease, nor of growth characteristics, appearance, or resistance towards antibiotics *in vivo*. There was no evidence indicating the patient suffered from a mixed infection, and multiple plate sweeps all pointed to a single genetic clone. This indicates that there are environments or conditions where the full-length *abcZ* is not a prerequisite. It is possible that the truncated CDS *abcZ-2* is expressed and functional. On the other hand, the presumptive TTG start codon has no obvious ribosomal binding site (RBS) in the vicinity, and there are also no better start codons within reasonable distance. This could indicate that *abcZ-2* is not expressed at all.

The precise function of the ABC transporter that *abcZ* encodes is unknown, but most likely it is of the exporter type. Typically, exporters are less substrate specific than importers. Thus, if an exporter is compromised it is more probable that the function can be maintained through overexpression of other exporters than if the same happened to an importer. Similarly, it has been shown in *E. coli* that deletions of *dedA*-family genes *yghB* and *ygjA* can be compensated by increasing the expression of another efflux pump-encoding gene, *mdfA* [27–29]. The following evidence can support this being the case in the ∆abcZ strain: I) A substrate-binding domain protein was not found encoded in the same or neighbouring operons. II) The DedA protein has a predicted 4–6 transmembrane helices (TMH), which matches that of exporters. Importers usually have more TMHs. There are DedA proteins modelled to have an α-helical protein fold located in the inner membrane [30], including the *E. coli* DedA homologue constructed by the AlphaFold2 (Accession ID: P0ABP6). This is supported by the fact that most inner-membrane proteins are α-helical, while beta-barrels are the most common fold in the outer membrane [31]. III) The SwissModel Structural assessment does indicate that further optimizations could improve the sidechains and fine tune the model, however, the overall topology does seem likely. The assessment also supports that DedA is a membrane protein. IV) An in-depth evolutionary study concluded that DedA superfamily consist of 5–8 transmembrane helices with an ion-coupled function [32]. There is an ongoing discussion regarding whether ABC transporters dimerize in nature or if this is a laboratory artefact that occurs during crystallization (33). When one NBD is knocked out like in the ∆*abcZ* strain, it is possible that the DedA protein binds to monomeric AbcZ-2 and that the complex remains functional. Another possibility is that AbcZ-2 dimerizes and binds with dimerized DedA. Further laboratory work is needed to conclude which of these alternatives are more likely under physiological conditions.

## Supplementary Data

Supplementary material 1

Supplementary material 2
