# Supplementary figures and images for: Naturally occurring Neisseria gonorrhoeae can have large deletions in housekeeping gene abcZ, making them untypable with multilocus sequence typing
Source: Microb Genom. 2022 Sep 22;8(9):mgen000889. doi: 10.1099/mgen.0.000889 (PMC9676028; doi:10.1099/mgen.0.000889)

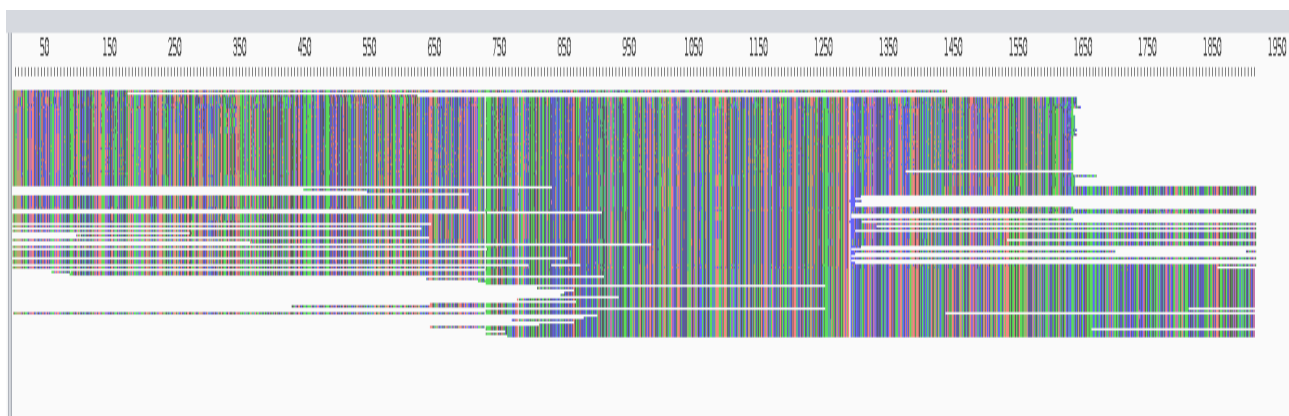

Supplement: Supplementary material 1 [file mgen-8-889-s001.pdf]
