# Supplementary material for: Naturally occurring Neisseria gonorrhoeae can have large deletions in housekeeping gene abcZ, making them untypable with multilocus sequence typing
Source: Microb Genom. 2022 Sep 22;8(9):mgen000889. doi: 10.1099/mgen.0.000889 (PMC9676028; doi:10.1099/mgen.0.000889)
Supplement: Supplementary material 2 [file mgen-8-889-s002.pdf]

```
CLUSTAL O(1.2.4) multiple sequence alignment

                                                                  Walker A
659717_Abc-Z1      MNILSVENASFAVGHVALLDKTSFQLDSGEKVGLIGRNGAGKSSFLKILTGVQKLDDGQI 60
659717_Abc-Z2      ------------------------------------------------------------ 0
48638              MNILSVENASFAVGHVALLDKTSFQLDSGEKVGLIGRNGAGKSSFLKILTGVQKLDDGQI 60

                            Q-loop
659717_Abc-Z1      IVQNNLKIVYVPQESFFDKDATVFARVGG------------------------------- 89
659717_Abc-Z2      ------------------------------------------------------------ 0
48638              IVQNNLKIVYVPQESFFDKDATVFDTVAEGLGEIRDLLRRYHHVSHELENGSSELLLKEL 120

                                                        Signature          Walker B
659717_Abc-Z1      ------------------------------------------------------------ 89
659717_Abc-Z2      ------------------------------------------------------------ 0
48638              NELQLEIEAKDGWKLDAAVKQTLGELGLPENEKIGNLSGGQKKRVALAQAWVQKPDVLLL 180

                      D-loop
659717_Abc-Z1      ------------------------------------------------------------ 89
659717_Abc-Z2      ----------------------------------------------------------MI 2
48638              DEPTNHLDIDAIIWLENLLKAFEGSLVVITHDRRFLDNIATRIVELDRGILRSYPGSFSK 240

659717_Abc-Z1      ------------------------------------------------------------ 89
659717_Abc-Z2      RTQPYLQELAVEAEHNRLFDKFHAQEEAWIRKGIEARRTRNEGRVRRLEELRRQRAERRN 62
48638              YSEKKAQELAVEAEHNRLFDKFHAQEEAWIRKGIEARRTRNEGRVRRLEELRRQRAERRN 300

                                                                      Walker A
659717_Abc-Z1      ------------------------------------------------------------ 89
659717_Abc-Z2      VQGQVNFKLDSGKKSGKIIAELEHASFAYDDKVIMDKFSAILQRGDKIGLIGPNGIGKTT 122
48638              VQGQVNFKLDSGKKSGKIIAELEHASFAYDDKVIMDKFSAILQRGDKIGLIGPNGIGKTT 360

                                            Q-loop
659717_Abc-Z1      ------------------------------------------------------------ 89
659717_Abc-Z2      FLKLILGELQPTYGRIRIGSKQEVAYFDQFRSALNENDTVFYTLGQGNDYVEVGGKKKHV 182
48638              FLKLILGELQPTYGRIRIGSKQEVAYFDQFRSALNENDTVFYTLGQGNDYVEVGGKKKHV 420

                                 Signature                   Walker B  D-loop
659717_Abc-Z1      ------------------------------------------------------------ 89
659717_Abc-Z2      MSYLEDFLFPPARAQSPVSSLSGGERNRLLLAKLFTRPANILVLDEPTNDLDIDTQELLE 242
48638              MSYLEDFLFPPARAQSPVSSLSGGERNRLLLAKLFTRPANILVLDEPTNDLDIDTQELLE 480

659717_Abc-Z1      ------------------------------------------------------------ 89
659717_Abc-Z2      DLLRDYQGTVFLVSHDRMFLDNVITQSIVFEGQGRLKEYIGGYQDYIDAKSRENKIQTAS 302
48638              DLLRDYQGTVFLVSHDRMFLDNVITQSIVFEGQGRLKEYIGGYQDYIDAKSRENKIQTAS 540

659717_Abc-Z1      ------------------------------------------------------------ 89
659717_Abc-Z2      APKASDVEPAKEKPKANRTVKLSYKEQRELDALPDEIAALETEQAEINAQLSDPGIFKDY 362
48638              APKASDVEPAKEKPKANRTVKLSYKEQRELDALPDEIAALETEQAEINAQLSDPGIFKDY 600

659717_Abc-Z1      -------------------------------------- 89
659717_Abc-Z2      EKAGALQNRAEEIEMLLLEKLERWELLETKQNGNAV 398
48638              EKAGALQNRAEEIEMLLLEKLERWELLETKQNGNAV 636
```
